# Evaluation of fetal acidemia during delivery using the conventional 5-tier classification and Rainbow systems

**DOI:** 10.1371/journal.pone.0287535

**Published:** 2023-06-23

**Authors:** Shoichi Magawa, Shintaro Maki, Masafumi Nii, Mizuki Yamaguchi, Yuya Tamaishi, Naosuke Enomoto, Sho Takakura, Kuniaki Toriyabe, Eiji Kondo, Tomoaki Ikeda

**Affiliations:** Department of Obstetrics and Gynecology, Mie University Faculty of Medicine, Mie, Japan; The Aga Khan University, PAKISTAN

## Abstract

The association between prepartum time-series fetal heart rate pattern changes and cord blood gas data at delivery was examined using the conventional 5-tier classification and the Rainbow system for 229 female patients who delivered vaginally. They were classified into three groups based on the results of umbilical cord blood gas analysis at delivery. The fetal heart rate pattern classifications were based on analysis of measurement taken at 10-min intervals, beginning at 120 min pre-delivery. The relationship between fetal heart rate pattern classification and cord blood pH at delivery changed over time. The 5-tier classification at each interval increased before delivery in the Mild and Severe groups compared with the Normal group. No significant differences were observed between acidemia groups. The Rainbow classification showed a significant differences between the acidemia groups at each interval, particularly during the prepartum period. A relationship between classification and outcome was evident before delivery for both the 5-tier classification and Rainbow system.

## Introduction

Cardiotocography (CTG) is a method of monitoring fetal status by continuously measuring fetal heart rate (FHR) and uterine contractions that was introduced into clinical practice more than 50 years ago for assessing fetal tolerance to hypoxic stress associated with labor and delivery [1–6]. Continuous FHR monitoring remains the most useful tool for screening for fetal abnormalities so that an appropriately timed medical intervention can be initiated and hypoxic injury during delivery can be avoided [7]. However, FHR monitoring is highly sensitive and has low specificity. This has caused an increased rate of cesarean sections and surgical deliveries and the failure to decrease the incidences of infant mortality and cerebral palsy [8].

Therefore, developing an accurate method of assessing fetal acidemia for the hypoxic load associated with intermittent uterine contractions during labor and delivery is necessary. Furthermore, persistent hypoxemia during labor may cause a gradual decrease in fetal cardiac output, leading to fetal hypotension and hypoperfusion, which may result in hypoxic-ischemic brain injury [9–15].

Moreover, this progressive worsening of fetal hypoxemia during the labor is reflected by changes in baseline FHR and deeper FHR deceleration [16].

It was hypothesized that the change in fetal acidemia could be more precisely evaluated using FHR monitoring, which includes the aforementioned physiological changes as evaluation items. Therefore, the conventional evaluation method was compared using the Trium® computerized 5-tier monitoring system every 10 min with the Rainbow system, which evaluates CTG 5-tier levels over time. In addition, the relation between these evaluation methods and umbilical cord arterial blood gas pH analysis at birth was assessed.

## Materials and methods

This was a retrospective cross-sectional study of patients who delivered vaginally at Mie University Hospital. This study included cases of singleton pregnancies that resulted in vaginal delivery at Mie University Hospital from January 2017 to December 2019. Inclusion criteria were maternal age of 18–45 years, singleton pregnancy, and FHR monitoring data from 120 min before delivery, which was stored as continuous data. Pregnancies that were complicated by maternal heart disease or fetal cyanotic heart disease that could have affected fetal cord blood gas analysis after birth were excluded. Cases of induction of labor and vacuum delivery were not excluded. Induction of labor was performed by intravenous oxytocin administration when the due date was exceeded (after 40 weeks and 0 days of gestation), when delivery was stopped (process of labor was arrested for > 2 hours despite transient progress after the start of labor), or labor did not start after membrane rupture. Vacuum delivery was performed when fetal dysfunction or delivery arrest was considered to have occurred as a result of the descent of the infant’s head (Station: +2 or lower) with the progress of labor and delivery. Vacuum delivery was performed three times or for a total suction time ≤ 10 minutes.

Group classification was based on umbilical cord blood gas pH analysis, with pH ≥ 7.2, 7.2 > pH > 7., and pH ≤ 7.1 defining the Normal, Mild, and Severe groups, respectively. The obstetrician promptly collected the umbilical cord blood gas from the umbilical cord artery after infant delivery. Umbilical cord arterial blood gas analysis was performed using the RAPID Point 500 Blood Gas Analyzer (Siemens Healthcare, UK). The pO_2_ value was not evaluated because the RAPID Point 500 cannot measure detailed pO_2_ values < 10 mmHg.

FHR was measured using external monitoring or a scalp electrode, uterine activity was assessed using tocodynamometry, and the Toitu MT 810 B fetal monitor was used for registration, at a paper speed of 3 cm/min. CTG data were continuously traced without a trace loss of 20 s. Trium CTG OnlineVR (GE HealthcareVR, Little Chalfont, UK and Trium Analysis Online GmbH, Munich, Germany) is a computer analysis system used to evaluate both the FHR and uterine contractions to quantify the CTG trace components, such as baseline FHR, acceleration, any deceleration, and baseline variability. It distinguishes the following types of deceleration: 1) late deceleration, which is a gradual decrease in the FHR (defined as an onset of deceleration to nadir of 30 s) associated with uterine contractions, where the nadir of deceleration occurs after the peak contraction. Late deceleration was considered severe when FHR decreased to > 15 beats per minute (bpm) below the baseline. 2) Variable deceleration, which is an abrupt decrease in the FHR (defined as an onset of deceleration to a nadir of < 30 s) lasting 15 s to < 2 min. The severity of variable deceleration was defined according to the duration and lowest FHR. 3) Prolonged deceleration, which was defined as a decrease in FHR of ≤ 15 bpm, lasting 2 min to < 10 min. Prolonged deceleration with a nadir < 80 bpm was considered severe deceleration.

Based on this, the mean FHR classification was calculated for 10 bpm [17]. The normal baseline was defined as 110–160 bpm. Long-term variability was considered a quantitated description of the oscillations’ amplitude near the baseline FHR, and was defined as the standard deviation of the smoothed FHR signal, excluding deceleration and regions with poor signal quality. The “normal” variability was frequently the standard deviation of 6–25 bpm. Additionally, regarding the level categories, the baseline of each section, deceleration type, baseline, variability, and classification should be considered [18]. The sections were categorized every 10 min, and the highest level was adopted when multiple levels were mixed in the section.

### Rainbow system

The Rainbow system is an FHR monitoring evaluation system implemented in Trium®. Its computer reading criteria are as previously described, and the system reads the data every 10 s for the 10 min before the reading point.

**Fig 1:**
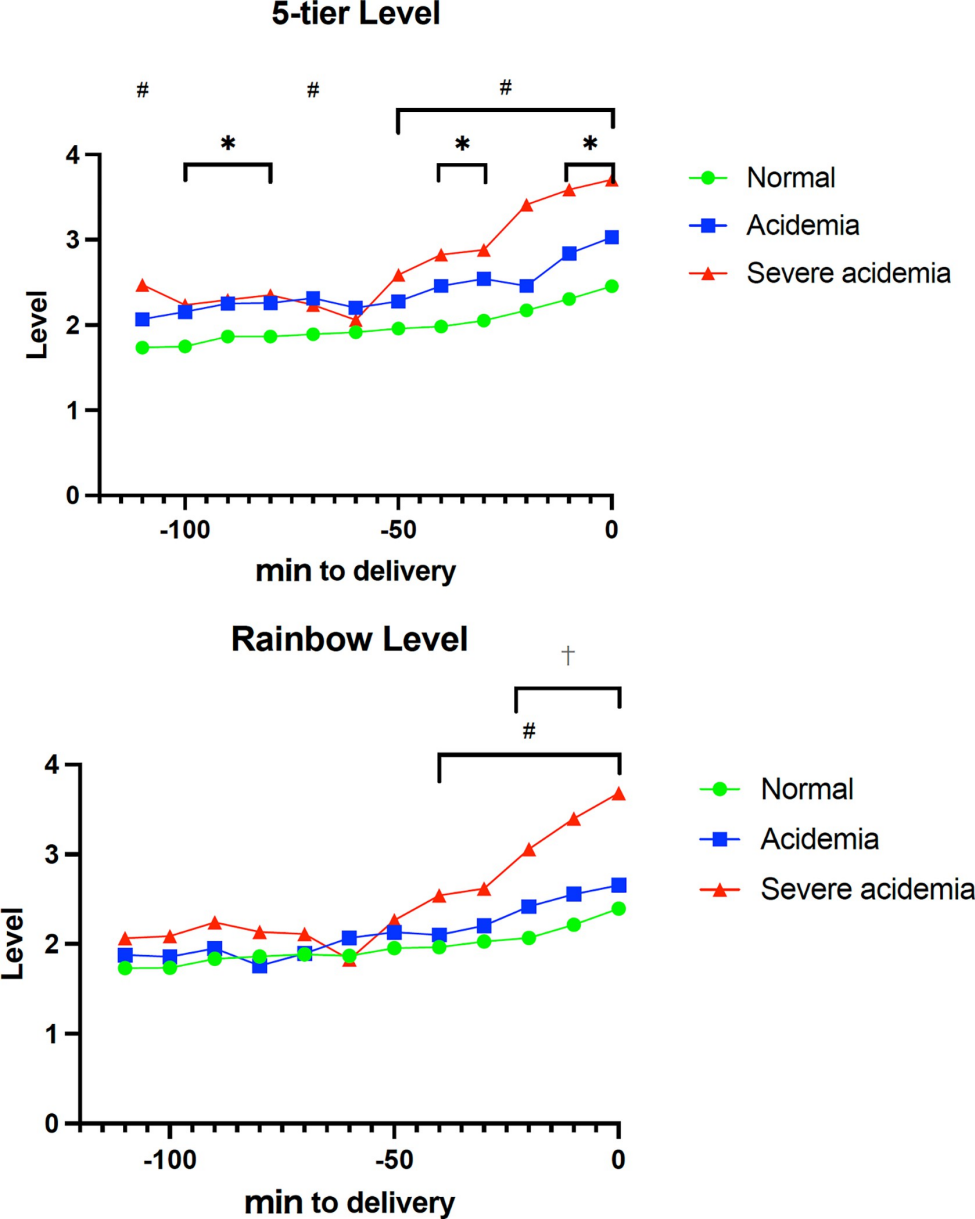
CTG levels of each group before delivery in the conventional 5-tier classification and Rainbow system. Circles indicate the level course of the Normal group, squares indicate that of the Mild group, and triangles indicate that of the Severe group. The transition was the average change in each group. #^,^ *, and † indicate the periods when there were significant differences between groups: # for Normal vs. severe, * for Normal vs. Mild, and † for Mild vs. Severe. A shows a group comparison of CTG levels using the conventional 5-tier classification. B shows a comparison of CTG levels using the Rainbow system.

Fig 1A and 1B shows the reading results as a color change at the top of the monitor. Specifically, the reading results are shown in five colors based on the classifications described above, and a sheet was created every 10 min. The FHR classification during each 10-min period was evaluated in terms of the time spent on each sheet. That is, level × duration (min)/10 was calculated for each level, and the sum was considered the level of that sheet.

### Statistical methods

Maternal and fetal backgrounds were compared between groups using the Kruskal–Wallis test. Between-group differences were evaluated using the Bonferroni post hoc test when significant differences were found using the Kruskall–Wallis test.

Changes in the conventional 5-tier classification and the Rainbow system classification were evaluated between groups every 10 min during the 120 min leading up to delivery using the Kruskal–Wallis test. However, when significant differences were found, between-group differences were evaluated using the Bonferroni post hoc test.

The Pearson chi-square test was also performed to compare the three groups in terms of the following factors: babies’ sexes, 5-min Apgar scores (<7 or not) and whether they were delivered via vacuum and induction or not. Finally, the Bonferroni method was used to adjust the *p*-values. In all tests, statistical significance was set at *p* < 0.05. Data were analyzed using the Statistical Package for the Social Sciences (SPSS) version 25 (IBM Corporation, Armonk, NY, USA).

### Ethical approval

This study was conducted in accordance with the principles of the Declaration of Helsinki, and its research protocol was approved by the Institutional Review Board of Mie University Hospital (IDH2021-232). The need for informed consent was waived, since this is a retrospective analysis. Patients who were eligible for this study had the opportunity to refuse to participate in the study by opting out.

## Results

In all, there were 1,369 singleton pregnancy deliveries at our hospital, of which 671 Cesarean section cases were excluded. Of the remaining 698 cases, 34 cases of maternal heart disease and 45 cases of fetal heart disease were excluded. Of the 619 remaining cases, 229 were included in the analysis after excluding those with no continuous data from 2 hours before delivery to delivery, and those with no umbilical artery blood data. Overall, 175, 37, and 17 patients were assigned to the Normal, Mild, and Severe acidemia groups, respectively. Table 1 presents the patients’ background and demographic data.

**Table 1:** Background information of cases and statistical analysis results.

				Kruskal–Wallis	Bonferroni		
	Normal (n = 175)	Mild (n = 37)	Severe (n = 17)		Normal versus Mild	Normal versus Severe	Mild versus Severe
Age	32.75 (29, 37)	33.21 (30, 38)	38.35 (35, 38)	0.043	1.000	0.040	0.262
BMI at Delivery	25.86 (22.72, 28.04)	25.23 (22.07, 27.56)	23.30 (21.22, 24.61)	0.043	1.000	0.043	0.316
Number of births	0.55 (0, 1)	0.56 (0, 1)	0.41 (0, 1)	0.745			
Gestation days	275.37 (271, 284)	273.16 (268, 281)	253.71 (261, 280)	0.374			
Newborn weight (g)	3053 (2772, 3352)	2977 (2754, 3314)	2769 (2448, 3006)	0.088			
Apgar 1 min	8.07 (8, 8)	7.41 (7, 8)	6.47 (5, 8)	< 0.001	0.003	< 0.001	0.140
Apgar 5 min	8.92 (9, 9)	8.46 (8, 9)	8.24 (8, 9)	0.001	0.034	0.010	0.971
pH	7.29 (7.16, 7.32)	7.16 (7.14, 7.19)	7.07 (7.04, 7.10)	< 0.001	< 0.001	< 0.001	0.515
BE	-5.78 (-7.38, -4.10)	-9.90 (-11.4, -8.2)	-12.85 (-15.5, -11.0)	< 0.001	< 0.001	< 0.001	0.936
pCO_2_	42.86 (38.85, 47.75)	51.90 (47.10, 58.20)	59.35 (49.30, 71.50)	< 0.001	< 0.001	< 0.001	0.922

Values are presented as mean (interquartile range).

BMI: body mass index, BE: base excess; pCO_2_

No significant differences were observed between the groups regarding the number of births, gestational days, and neonatal weight.

The following parameters showed significant differences between the Normal and Mild groups and the Normal and Severe groups: Apgar score (1 min) (*p* = 0.003, *p* < 0.001), Apgar score (5 min) (*p* = 0.034, 0.010), pH (*p* < 0.001, *p* < 0.001), base excess (BE) (*p* < 0.001, *p* < 0.001), and pCO_2_ (*p* < 0.001, *p* < 0.001). The following parameters showed significant differences between the Normal and Severe groups: age (*p* = 0.040) and BMI at delivery (*p* = 0.043). The proportions of patients with 5-min Apgar scores < 7 for each group are listed in Table 2. The Normal group had a significantly lower rate of 5-min Apgar score < 7 than the Mild and Severe groups. The distribution of induced deliveries for each group is shown in Table 2. There were no significant differences in induction among groups. Additionally, no significant differences were observed among the groups in terms of the sex of the newborns. However, significant differences were found between the Normal (12.0%) and Severe (47.1%) groups in terms of vacuum delivery use (Table 2).

**Table 2:** Comparison of sex and whether vacuum delivery was performed.

		Normal		Mild		Severe	
		Number	%	Number	%	Number	%
Vacuum extraction	No	154^#^	88.0	28	75.7	9^#^	52.9
	Yes	21^#^	12.0	9	24.3	8^#^	47.1
		175	100	37	100	17	100
		Normal		Mild		Severe	
		Number	%	Number	%	Number	%
Sex	male	84	48	15	40.5	12	70.6
	female	91	52	22	59.5	5	29.4
		175	100	37	100	17	100
		Normal		Mild		Severe	
		Number	%	Number	%	Number	%
APGAR 5 min	≧ 7	173^a^	98.9	34^b^	91.2	15^b^	88.2
	< 7	2^a^	1.1	3^b^	8.8	2^b^	11.8
		175	100	37	100	17	100
		Normal		Mild		Severe	
		Number	%	Number	%	Number	%
Induction	No	121	69.1	24	64.9	13	76.5
	Yes	54	30.9	13	35.1	4	23.5
		175	100	37	100	17	100

#: indicates a significant difference between groups

a,b: Each subscript letter denotes a subset of Experimental Condition categories whose column proportions do not differ significantly from each other at level .05".

Fig 1A and 1B shows the results of the conventional 5-tier classification and the Rainbow system classification. No significant differences between the Mild and Severe groups were observed in the conventional 5-tier classification. Significant differences were observed between the Normal and Mild groups (marked by * in Fig 1A) and the Normal and Severe groups (marked by # in Fig 1A and 1B) with the conventional 5-tier classification. Specifically, significant differences between the Normal and Mild groups were found in the 110–100-min (*p* = 0.016), 100–90-min (*p* = 0.040), 90–80-min (*p* = 0.026), 50–40 min (*p* = 0.006), 40–30-min (*p* = 0.003), 20–10-min (*p* = 0.002) and 10–0-min (*p* = 0.002) prepartum periods with the conventional 5-tier classification. In contrast, significant differences between the Normal and Severe groups were found in the 120–110-min (*p* = 0.010), 80–70-min (*p* = 0.027), 60–50-min (*p* = 0.029), 50–40-min (*p* = 0.010), 40–30-min (*p* = 0.020), 30–20-min (*p* < 0.001), 20–10-min (*p* < 0.001), and 10–0-min (*p* < 0.001) prepartum periods with the conventional 5-tier classification. Furthermore, the Rainbow system showed no significant differences between the Normal and Mild groups. Specifically, significant differences were found between the Normal and Severe groups in the 50–40-min (*p* = 0.007), 40–30-min (*p* = 0.009), 30–20-min (*p* < 0.001), 20–10-min (*p* < 0.001), and 10–0-min (*p* < 0.001) prepartum periods. In contrast, significant differences were found between the Mild and Severe groups (described as † in Fig 1B) in the 30–20-min (*p* = 0.019), 20–10-min (*p* = 0.004), and 10–0-min (*p* = 0.002) prepartum periods with the Rainbow system.

## Discussion

The results of this study showed sustained significant differences between the Mild and Severe groups in the Rainbow system assessment from 30 min before delivery. However, no significant differences in the conventional 5-tier classification were observed between the Normal and Mild groups. This is an interesting result, given that a significant difference was observed in the conventional 5-tier classification between the Normal and Mild groups and the Normal and Severe groups, but not between the Mild and Severe groups.

FHR monitoring is the most common method for evaluating fetal health during delivery. It can be used to evaluate fetal health almost in real-time via analysis of heart rate changes, and is used in approximately 90% of deliveries in the United States, Canada, and other countries [19, 20]. However, no consensus exists regarding the optimal method of interpreting FHR results. Guidelines using 3-or 5-tier classification systems have been proposed in many countries [18, 21–23].

Some reports have adopted umbilical arterial blood gas pH < 7.1 as a criterion for significant acidosis in newborns [24]. However, the criteria vary among reports, with some reporting that a < 10% value indicates pH < 7.1 [25], and others that a 2.5% value is equivalent to pH 7.08 [26].

During the onset of labor, current perinatal care aims to assess whether the fetus can tolerate intermittent hypoxic exposure associated with uterine contractions and to provide medical intervention at the appropriate time to avoid irreversible damage. Considering the abovementioned reports, delivering the fetus before reaching an umbilical artery blood gas pH < 7.1 is desirable.

Various methods exist for conventional 3- and 5-tier classifications, and which of them is the most appropriate remains a subject of debate. The 3-tier classification was recommended by the 2008 National Institute of Child Health and Human Development, primarily because it is simpler than the conventional 5-tier classification and the accumulated evidence [27, 28].

The conventional 5-tier classification, which is used in Japan, some regions of the United States, and France, has a moderate interobserver agreement rate, similar to that of the conventional 3-tier classification [29, 30], It is also superior in detecting fetal acidemia during delivery [31, 32], and enables chronological evaluation of fetal acidemia progression [33].

Therefore, to evaluate the accuracy of time-series assessments using the conventional 5-tier classification, this study retrospectively compared the effectiveness of the conventional 5-tier classification and that of the Rainbow system by Trium®.

This study’s results showed that the computerized conventional 5-tier classification consistently showed significant differences between the Normal and Severe groups at 50 min before delivery and between the Normal and Severe groups at 20 and 40–30 min before delivery. However, the Rainbow system showed significant differences between the Severe and Normal groups at 50 min before delivery and between the Severe and Mild groups at 30 min before delivery. The vacuum delivery rate was higher in the Severe group than that in the Normal group. Therefore, an increase in the CTG level 10 min before delivery would be expected, considering the fetal bradycardia associated with vacuum delivery. However, vacuum delivery did not take longer than 10 min in any of the cases in this study, and no significant differences between the Severe and Mild groups in the percentage of vacuum deliveries performed were found. Therefore, it was hypothesized that the significant systematic differences in prepartum levels might reflect worsening fetal status, indicating the hypoxic addition associated with delivery. Additionally, this study’s results showed that the conventional 5-tier classification could distinguish two different levels of acidemia from the Normal group. In contrast, the Rainbow system could distinguish more severe acidemia from the Normal group continuously from 50 min before delivery, and mild and severe acidemia from 30 min before delivery. These results suggest that the conventional 5-tier classification is superior in detecting acidemia in the controls and that the Rainbow system is superior in in its ability to distinguish more severe acidemia from milder acidemia.

Although the Apgar score is a direct indicator of neonatal health [34] and does not directly reflect the results of cord blood gas analysis [35], it is important to evaluate these two tests together for a comprehensive evaluation of a newborn’s health.

In general, the 5 min-Apgar score is more sensitive than the 1-minute Apgar score for assessing neonatal prognosis, and a 5 min-Apgar score < 7 is associated with neurological disorders, gastrointestinal and infectious disease morbidity, and neonatal death [36–39].

Even considering the distribution of cases with a 5 min-Apgar score <7, the number of cases was significantly increased at pH <7.2. The 5 min-Apgar score is related to the neurological prognosis of the neonate, and a combination of the conventional 5-level classification and the Rainbow system may be effective in ensuring appropriately timed deliveries.

Notably, the waveform interpretation was performed using a computer in this study. The authors evaluated the agreement between obstetricians and Trium® readings in a previous study. Therefore, it was concluded that the computerized conventional 5-tier FHR monitoring reading using Trium® would show substantial agreement with the obstetrician’s reading and could be implemented in clinical practice [40].

In this study, a similar system was used to perform the conventional 5-tier classification and Rainbow system evaluations, and level changes in the Rainbow system, assessed clinically in real-time, may be able to more accurately identify fetal acidemia that requires medical intervention.

In conclusion, this study evaluated the conventional 5-tier classification based on FHR monitoring and the Rainbow system’s 5-tier classification (Trium®). The Rainbow system sensitively assessed fetal acidemia based on pH < 7.1. However, future studies are needed to improve the accuracy of FHR monitoring tracings and computer interpretations.

### Limitations of the study

This study had some limitations. First, the computer interpretation results were not evaluated. As mentioned in a previous study, when tracing loss occurs in FHR monitoring with bradycardia, the presence of bradycardia in that area cannot be detected or properly evaluated [40]. Therefore, excluding cases with partial tracing loss during FHR monitoring is necessary. Second, cases of acidemia were evaluated to determine whether they were caused by hypoxia during delivery. Determining presence or absence of neonatal encephalopathy could have enabled the recruitment of cases of hypoxic exposure at delivery to more appropriately assess hypoxia-associated impairment at delivery [41]; however, this assessment would have been difficult to conduct for several reasons; for instance, postnatal magnetic resonance imaging evaluation was not properly performed. In addition, this study did not consider risks other than maternal and fetal cardiac disease. Future observational studies are needed to assess other risks and to determine whether the monitoring process at delivery differs when these risks are present.
